# Effect of Dietary Patterns on Maternal Body Composition and Bone Mineral Density During Three Trimesters in Chinese Pregnant Women: A Cross-Sectional Study

**DOI:** 10.3390/nu17122021

**Published:** 2025-06-17

**Authors:** Jiajun Liu, Zhen Qin, Ziwei Xi, Yalin Zhou, Yajun Xu

**Affiliations:** 1 School of Public Health, Peking University, No. 38 Xueyuan Road, Beijing 100083, China; jiajunliu@stu.pku.edu.cn (J.L.); qinzhen@stu.pku.edu.cn (Z.Q.); 2010306240@stu.pku.edu.cn (Z.X.); zylyingyang@163.com (Y.Z.); 2 Beijing Key Laboratory of Toxicological Research and Risk Assessment for Food Safety, Peking University, No. 38 Xueyuan Road, Beijing 100083, China; 3 PKUHSC—China Feihe Joint Research Institute of Nutrition and Healthy Lifespan Development, No. 38 Xueyuan Road, Beijing 100083, China

**Keywords:** pregnancy diet, body composition, bone mineral density, dietary assessment indices

## Abstract

**Background/Objectives**: This study aims to assess dietary quality among Chinese pregnant women across three gestational trimesters using different assessment indices while investigating the relationship between dietary patterns and longitudinal alterations in maternal body composition parameters and bone mineral density (BMD) during pregnancy. **Methods**: A total of 556 healthy pregnant women were recruited. Dietary intake was assessed utilizing a semi-quantitative food frequency questionnaire (FFQ). Diet quality was evaluated through three indices: the Dietary Balance Index for Pregnant Women (DBI-P), the Dietary Inflammatory Index (DII), and the Eastern Health Diet Index (EHDI). Multiple linear regression models and mediation analyses were constructed to elucidate the relationships between dietary indices, body composition parameters, and BMD. **Results**: In the first trimester, excessive dietary intake was associated with increased maternal fat mass but reduced BMD, while insufficient intake correlated with declines in muscle mass, water compartments, and inorganic salt levels. Pro-inflammatory diets further exacerbated reductions in non-fat body composition, including protein and muscle mass. By the second trimester, low-quality diets continued to negatively affect muscle mass and water balance, whereas no significant dietary effects on body composition or BMD were observed in the third trimester. Mediation analyses revealed that body composition partially mediated the relationship between dietary imbalance and reduced BMD. **Conclusions**: Unbalanced, pro-inflammatory, and low-quality diets during early-to-mid pregnancy contribute to adverse changes in maternal body composition and bone health, especially in the first and second trimesters, with the DBI-P index demonstrating superior applicability for assessing dietary impacts in Chinese pregnant women.

## 1. Introduction

Maternal nutrition holds critical importance associated with both maternal and fetal health outcomes. A nutritionally balanced gestational diet mitigates the risks of complications such as gestational diabetes mellitus (GDM), pre-eclampsia, and excessive weight gain [1]. Moreover, it simultaneously supports optimal fetal growth and reduces the risk of obesity and metabolic dysregulation in the offspring [2]. Maternal body composition parameters (encompassing fat mass, muscle mass, and fluid balance) and bone mineral density (BMD) undergo significant alterations during different pregnancy stages, characterized by increases in total body water and fat, alongside a transient decline in BMD [3,4]. Notably, maternal dietary patterns exert bidirectional influences on the two interconnected physiological systems. Excessive adiposity accumulation and reduced muscle mass correlate with insulin resistance and cardiometabolic dysfunction [5], while pregnancy-associated BMD reductions—driven by fetal skeletal mineralization demands—may predispose maternal osteoporosis risk in later life, particularly when compounded by inadequate nutrient intake [6,7]. Consequently, systematic evaluation of trimester-specific dietary impacts on maternal body composition parameters and BMD remains imperative.

While diets of Chinese populations exhibit notably complex dietary structures and ingredient diversity, cultural and physiological dietary assessment tools are essential to objectively evaluate prenatal dietary influences on maternal–fetal outcomes [8]. A comparison between three different dietary patterns is presented in Table 1. The Dietary Balance Index for Pregnant Women (DBI-P) was designed to quantify the adherence to Chinese Dietary Guidelines while identifying imbalances between nutrition adequacy and excess [9]. Prior applications have linked DBI-P to pregnancy complications and neonatal outcomes [8,10,11]. The Dietary Inflammatory Index (DII), widely validated across populations, quantifies the potential inflammation of dietary patterns and has been adapted for Chinese pregnant women [12]. In contrast, the Eastern Health Diet Index (EHDI) evaluates dietary quality based on Eastern healthy dietary patterns, emphasizing diverse vegetables, fruits, seafood, dairy, beans, and less oil preparation, which is recommended by Chinese Dietary Guidelines [13].

Though emerging epidemiological studies have linked diet and BMD during pregnancy [14,15], evidence connecting diet assessed by DBI-P, DII, and EHDI to body composition parameters and BMD, especially the dynamic relationship, remains sparse. Utilizing three dietary indices, our research aimed to explore the relationship between diet, BMD, and body composition parameters while considering the mediating effect of body composition parameters on the relationship between diet and BMD.

## 2. Materials and Methods

### 2.1. Study Population

The study utilized a prospective cross-sectional design, enrolling 680 pregnant women from Changping Maternal and Child Health Hospital in Beijing, China between April 2018 and December 2019. Participants were required to meet the following inclusion criteria: (1) pregnant women aged 18 years or older; (2) absence of diagnosed chronic diseases, including cardiovascular, hepatic, or renal disorders; (3) no prolonged use of antibiotics or other medications known affect BMD or body composition; (4) singleton pregnancy with no prior history of pregnancy termination. Exclusion criteria included: (1) nonadherence to study protocols and (2) insufficient data for primary outcome measures.

**Figure 1:**
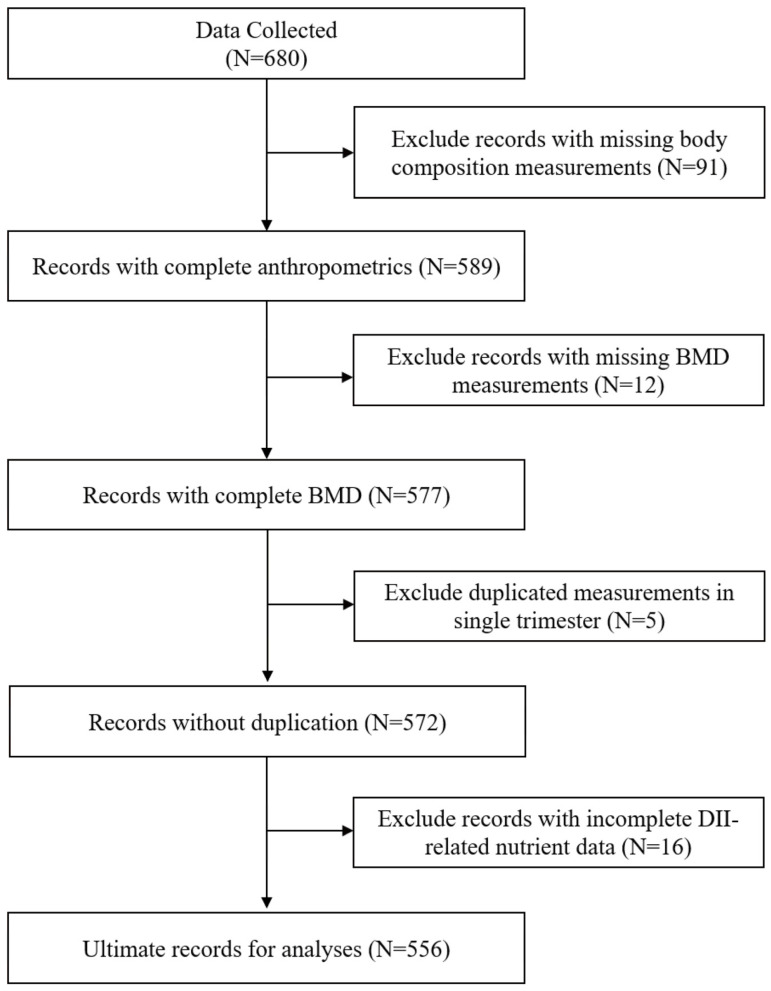
Flowchart of participants in the analyses.

Consequently, 556 participants met eligibility requirements (Figure 1) and completed the following assessments, including semi-quantitative food frequency questionnaires (FFQ), BMD, and body composition evaluations during the first, second, and third trimesters. Trained researchers informed the participants about the objectives of the investigation, provided fundamental details about the study, and obtained individual informed consent. Precise, visual, scaled, and weighted food safeguards were employed to mitigate the recall bias of self-reported dietary data. Ethical approval for the study protocol was obtained from the Medical Ethics Committee of Peking University.

### 2.2. Dietary Assessment

Dietary intake was evaluated utilizing a semi-quantitative FFQ, adapted from a previously validated FFQ [16]. Participants were instructed to report both the frequency and portion sizes of consumption across 18 food categories, including refined cereals, whole grains, legume and legume products, dark-colored vegetables, light-colored vegetables, dark-colored fruits, light-colored fruits, poultry, meat and meat products, fish and aquatic products, eggs, dairy products, fungi and algae, nuts, 100% fruit/vegetable juices, honey and candies, pastries and sugar-sweetened beverages. Daily intake quantities for each food group were calculated by integrating self-reported frequency and portion size data. Total energy and macronutrient intake were derived from the Chinese Food Composition Table [17]. For micronutrients and bioactive compounds, flavonoids, proanthocyanidin, vitamin D, and calcium (both from food and dietary supplements) were estimated utilizing the United States Department of Agriculture (USDA) database [18]. Additionally, isoflavone intake was specifically assessed with reference to the Hong Kong database [19].

### 2.3. Dietary Assessment Indices

Three validated dietary indices were employed to evaluate dietary patterns.

#### 2.3.1. Dietary Balance Index for Pregnancy (DBI-P)

The DBI-P, adapted from prior methodologies and aligned with the Chinese Dietary Guidelines (2022) [20], evaluates adherence to recommended intakes across seven primary categories and ten subcategories, including cereals and tubers, vegetables, fruits, dairy and dairy products, legumes and legume products, poultry and livestock meats, aquatic products, eggs, dietary diversity, and water intake. Scores were assigned as positive scores (exceeding recommendations), negative scores (below recommendations), and zero scores (adherence to recommendations). The magnitude of deviation from recommendations determined individual item scores. Utilizing aggregated scores of various DBI-P categories, three indicators were derived: High Bound Score (HBS), calculated as the cumulative sum of positive scores, quantifies excessive intake and ranges from 0 to 20; Low Bound Score (LBS), calculated as the cumulative sum of absolute negative scores, quantifies insufficient intake and ranges from 0 to 72; and Dietary Quality Distance (DQD), calculated as the cumulated sum of absolute scores, measures overall imbalance and ranges from 0 to 92.

#### 2.3.2. Dietary Inflammatory Index (DII)

DII has evaluated inflammatory potential utilizing 45 dietary components, nutrients, and bioactive substances. For each item, the Z-score was computed using a precise formula: Z-score = (daily intake − global average intake)/global standard deviation (SD). To minimize outlier effects, Z-scores were further converted into centered percentile scores and weighted by their inflammatory effect coefficients to generate DII_i_ (DII for individual items). The overall DII was calculated as the sum of all DII_i_. Positive DII values indicate pro-inflammatory diets, while negative values denote anti-inflammatory diets. Group comparisons were stratified by tertiles.

#### 2.3.3. Eastern Health Dietary Index (EHDI)

EHDI was designed to assess dietary alignment with energy contribution and the Eastern health diet pattern, which comprises 14 components: food diversity, whole grains, refined grains, vegetables, fruits, aquatic products, the ratio of poultry and livestock meats to aquatic products, dairy products, legumes and nuts, culinary oils, alcohol, salt, and added sugar.

Scoring for EHDI components follows a bidirectional system. Food groups recommended for increased consumption (e.g., whole grains, vegetables, fruits, aquatic products, dairy, legumes, and nuts) are assigned a score of 0 if intake meets or exceeds recommended thresholds and a score of 1 if intake is below recommendations. Conversely, food items advised for reduced consumption (e.g., refined grains, culinary oils, alcohol, salt, added sugar, and excessive poultry/livestock meats) receive a score of 0 for intakes below recommendations and a score of −1 for intakes above advised limits. By summing up the scores of the EHDI components, three indicators can be obtained. Positive Score (PS) reflects excessive intake of recommended food groups, calculated as the sum of positive values, with a range from 0 to 7. Negative Score (NS) assesses the deficits in dietary intake, calculated as the sum of the absolute values of negative values, with a range from 0 to 7. Total Score (TS) represents overall dietary quality, calculated as the sum of the absolute values of all components, with a range from 0 to 14.

### 2.4. Bone Mineral Density Assessment

BMD was assessed at the mid-right calcaneus, utilizing a CM-200 ultrasound bone densitometer (Furuno Electric, Nishinomiya City, Japan) operated by certified technicians. The instrument was initialized and calibrated by professional personnel before each assessment. Key BMD parameters, including broadband ultrasound attenuation (BUA), Z-scores, and T-scores were automatically calculated by the system.

### 2.5. Body Composition Assessment

Body composition parameters were evaluated utilizing a H-key350 human body composition analyzer (manufactured by Seehigher, Beijing, China). The measurements encompassed a comprehensive range of indicators, including fluid compartments (intracellular water, extracellular water, and total body water), tissue composition (protein content, inorganic salt levels, muscle mass (overall, right and left upper limbs, trunk, and right and left lower limbs)), and adiposity metrics (total body fat mass and regional fat distribution). All protocols adhered to standardized operational procedures to ensure measurement accuracy and reproducibility.

### 2.6. Covariates Assessment

The research collected characteristics of participants through a rigorous questionnaire, encompassing age, pre-pregnancy body mass index (BMI), educational attainment, ethnicity, average monthly household income, occupation, light physical activity status (including frequency and duration), daily sun exposure duration, and the presence of pregnancy-related complications (e.g., gestational diabetes mellitus (GDM)).

### 2.7. Statistical Analysis

Statistical analyses were conducted using R software (version 4.4.1, R Foundation for Statistical Computing, Vienna, Austria). A two-tailed significance threshold of 95% confidence intervals (CIs) was applied for all inferential tests. Continuous variables were presented as mean (sd), with comparison across groups via one-way analysis of variance (ANOVA). Categorical variables were reported as numbers (percentages), with analyses using Pearson’s chi-square tests. Bivariate correlations between dietary indices and food groups were quantified using Spearman’s rank correlation coefficients. To evaluate associations between dietary indices, body composition parameters, and bone mineral density (BMD), multivariable linear regression models were constructed, adjusting for covariates. These models were further extended to examine relationships between specific food groups, body composition parameters, and BMD. Moreover, mediation analysis was performed to assess the mediating effect of body composition on the relationship between dietary patterns and BMD, utilizing a nonparametric bootstrap approach. Sensitivity analyses compared effect estimates from unadjusted regression models with those adjusted for covariates to assess robustness against potential confounding

## 3. Results

### 3.1. Population Characteristics

A total of 556 pregnant women participated in the study, with an average age of 29.22 ± 4.01 years (mean ± SD) and an average pre-pregnancy BMI of 21.75 ± 3.25 kg/m^2^. Participants were stratified by trimester: 211 women in the first trimester, 185 in the second trimester, and 166 in the third trimester.

Demographic characteristics, dietary habits, body composition parameters, and BMD parameters across trimesters are summarized in Table 2. All body composition parameters demonstrated statistically significant differences (*p* < 0.05) among trimesters, while BMD and most dietary intake parameters showed minimal statistical significance, with the exception of egg consumption. Among demographic variables, GDM status exhibited statistically significant variations across trimesters.

### 3.2. Dietary Indices and Body Composition

**Figure 2:**
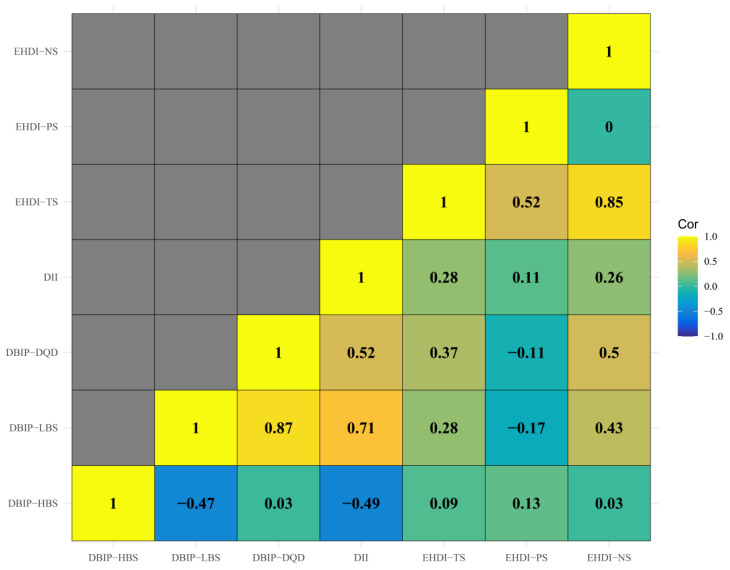
Correlation Matrix of Dietary Assessment Indices. Note: The heatmap illustrates Spearman’s correlation coefficients between the three dietary indices. DII shows strong correlations with DBIP-LBS, suggesting shared measurement of dietary assessment. Moderate correlations between DBIP-DQD, DII and EHDI-TS highlight distinct dietary aspects.

**Figure 3:**
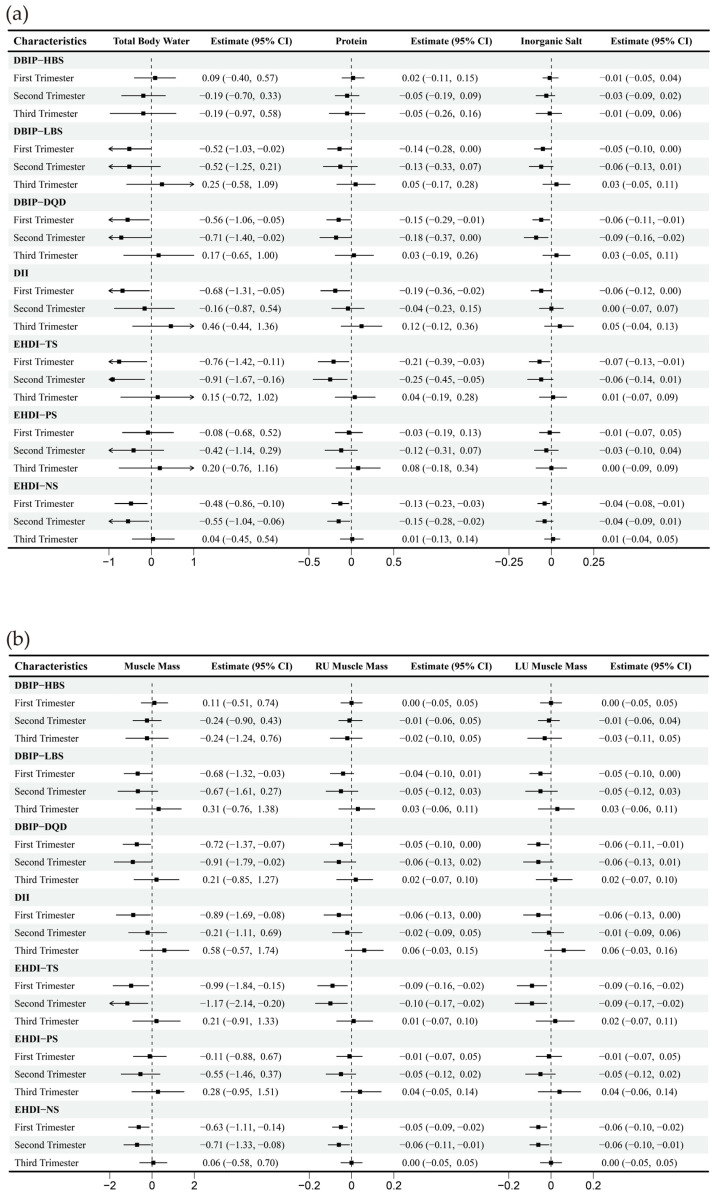

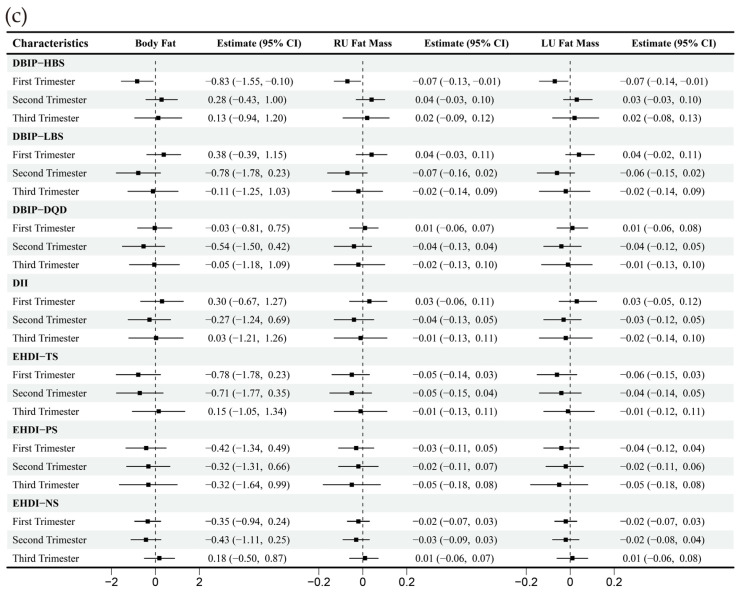
Multiple linear regression between dietary indices and body composition across trimesters. Note: Adjusted for age, gender, pre-pregnancy body mass index, education, income, gestational diabetes mellitus (GDM), light physical activity frequency and time, and sun exposure time. (**a**–**c**) Represents trimester-stratified regression results for associations between dietary indices and selected body composition parameters (encompassing fluid balance, muscle, and fat mass; full relevant associations are available in the Appendix A).

**Figure A1:**
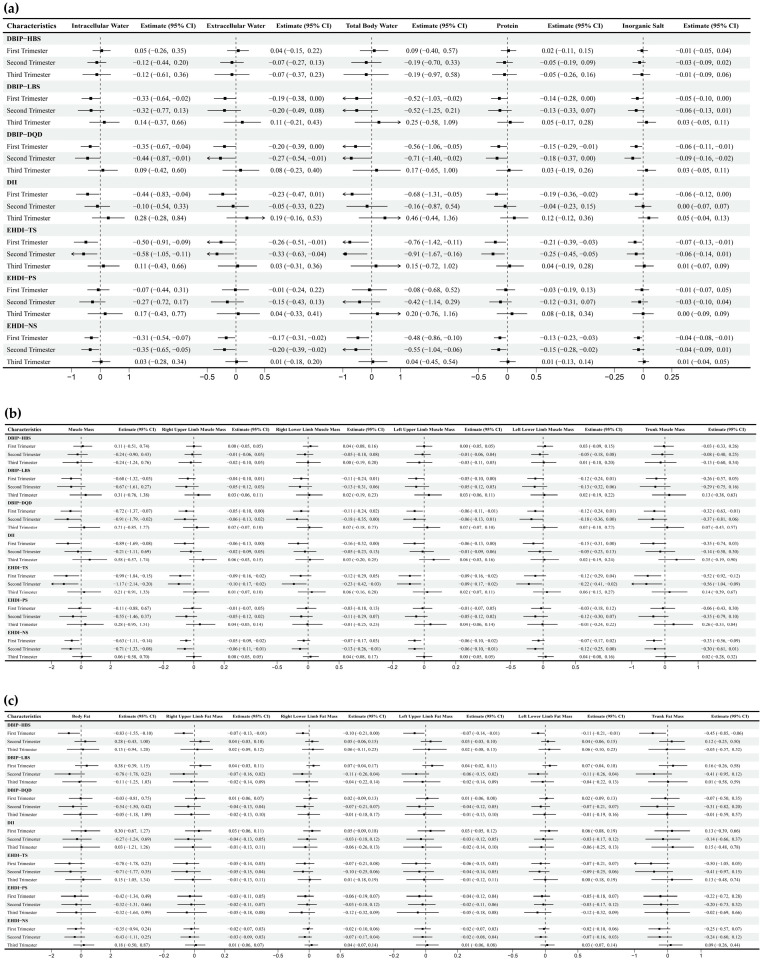
Multiple linear regression between dietary indices and body composition across trimesters. Note: Adjusted for age, gender, pre-pregnancy body mass index, education, income, gestational diabetes mellitus (GDM), light physical activity frequency and time, and sun exposure time. (**a**–**c**) Represents trimester-stratified regression results for associations between dietary indices and body composition parameters.

Strong correlations (Spearman’s correlation coefficient > 0.7) were observed between specific dietary indices: LBS and DQD, NS and TS, DII and LBS. Correlations among other dietary indices were comparatively weaker (Figure 2). The multivariate-adjusted associations between dietary indices and selected body composition are shown in Figure 3, and full relevant information is available in the Appendix A Figure A1. In the first trimester, HBS demonstrated an inverse correlation with fat mass, while LBS, DQD, DII, TS, and NS were mainly negatively correlated with other body composition parameters, including water, protein, inorganic salt, and muscle mass. During the second trimester, DQD, TS, and NS similarly correlated inversely with most non-fat body composition. No significant associations emerged in the third trimester.

**Figure 4:**
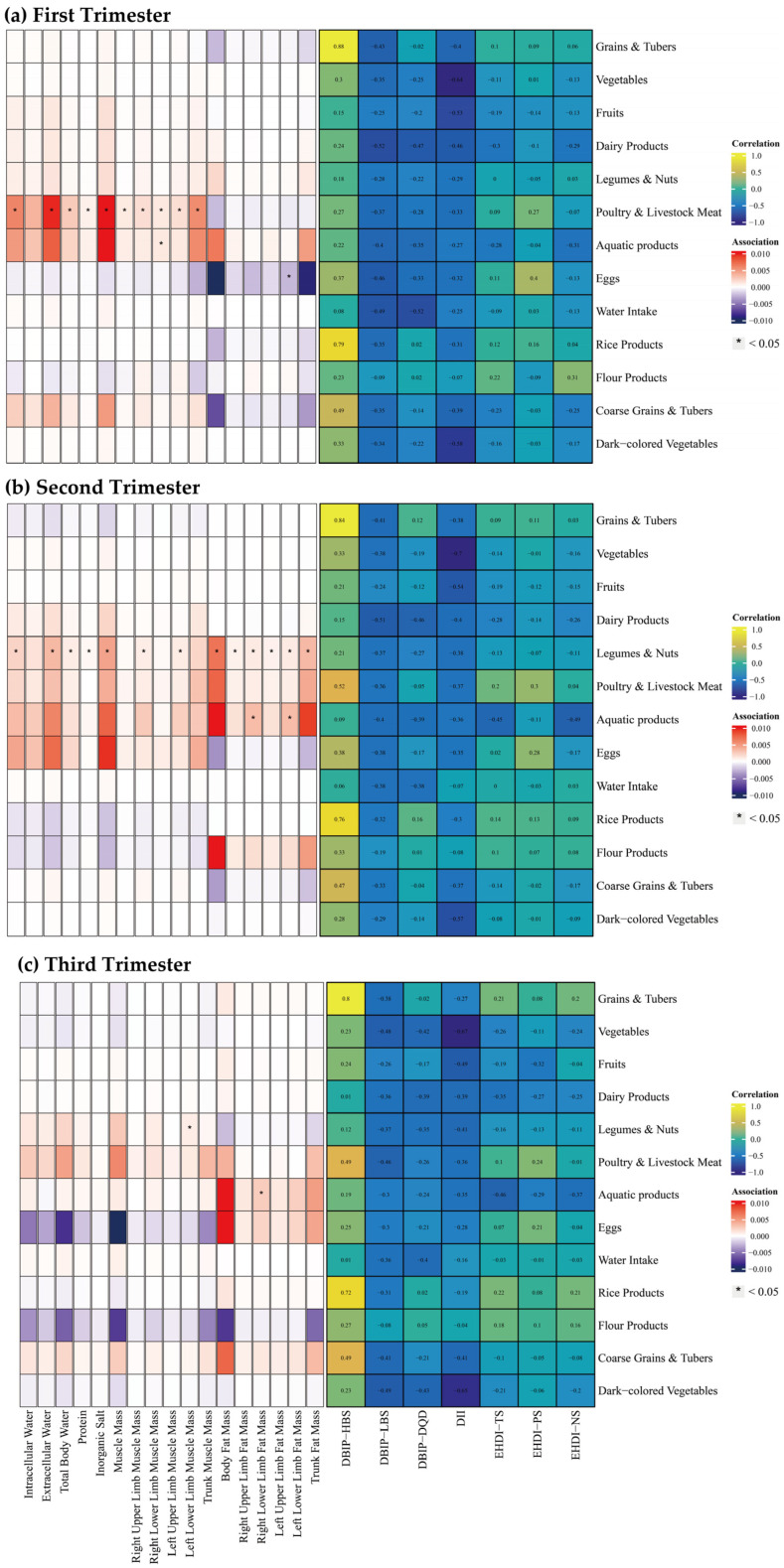
Spearman Correlation between Food Groups and Dietary Indices, and multiple linear regression between Food Groups and Body Composition across Trimesters. Note: The left panel displays the results of the multiple linear regression model, with Spearman correlation results in the right panel. The multiple linear regression model was adjusted for age, gender, pre-pregnancy body mass index, education, income, gestational diabetes mellitus (GDM), light physical activity frequency and time, sun exposure time. (**a**–**c**) Represents trimester-stratified results for correlations between food groups and dietary indices, and associations between food groups and body composition parameters.

Associations between food groups, dietary indices, and body composition parameters are illustrated in Figure 4. Food groups included grains and tubers, vegetables, fruits, dairy products, legumes and nuts, poultry and livestock meat, aquatic products, eggs, water intake, rice products, flour products, coarse grains and tubers, and dark-colored vegetables. Among dietary indices, HBS strongly correlated with the intake of grains and tubers, and rice products (Spearman’s correlation coefficient > 0.7), while other indices exhibited limited correlations.

During the first trimester, poultry and livestock meat intake was positively correlated with water intake, protein, inorganic salt, and muscle mass. Similarly, aquatic product intake was positively correlated with left upper limb muscle mass, whereas egg consumption was inversely correlated with left lower limb muscle mass. In the second trimester, intake of legumes and nuts indicated widespread positive correlations with body composition parameters. Aquatic product consumption was linked to increase in bilateral lower limb fat mass. During the third trimester, legumes and nuts intake was positively correlated with left lower limb muscle mass, while aquatic product intake was correlated with elevated right lower limb fat mass.

### 3.3. Dietary Indices and Bone Mineral Density

**Figure 5:**
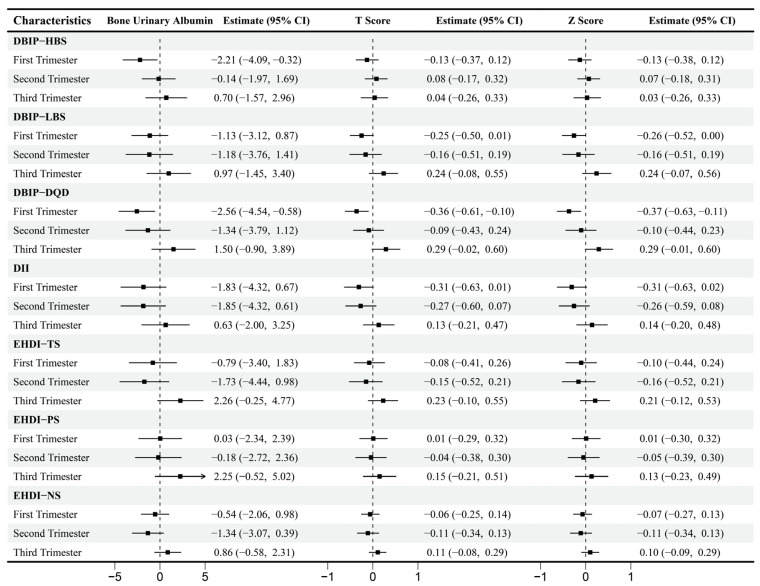
Multiple linear regression between Dietary Indices and Bone Mineral Density across Trimesters. Note: Adjusted for age, gender, pre-pregnancy body mass index, education, income, gestational diabetes mellitus (GDM), light physical activity frequency and time, sun exposure time.

Multivariable-adjusted correlations between dietary indices and BMD are presented in Figure 5. During the first trimester, HBS exhibited a significant negative correlation with BUA (β = −2.21, 95% CI [−4.09, −0.32]), while LBS negatively correlated with Z-score (β = −0.26, 95% CI [−0.52, 0.00]). DQD demonstrated a negative correlation with BUA (β = −2.56, 95% CI [−4.54, −0.58]), T-score (β = −0.36, 95% CI [−0.61, −0.10]) and Z-score (β = −0.37, 95% CI [−0.63, −0.11]). No significant correlations were observed between dietary indices and BMD during the second or third trimesters.

**Figure 6:**
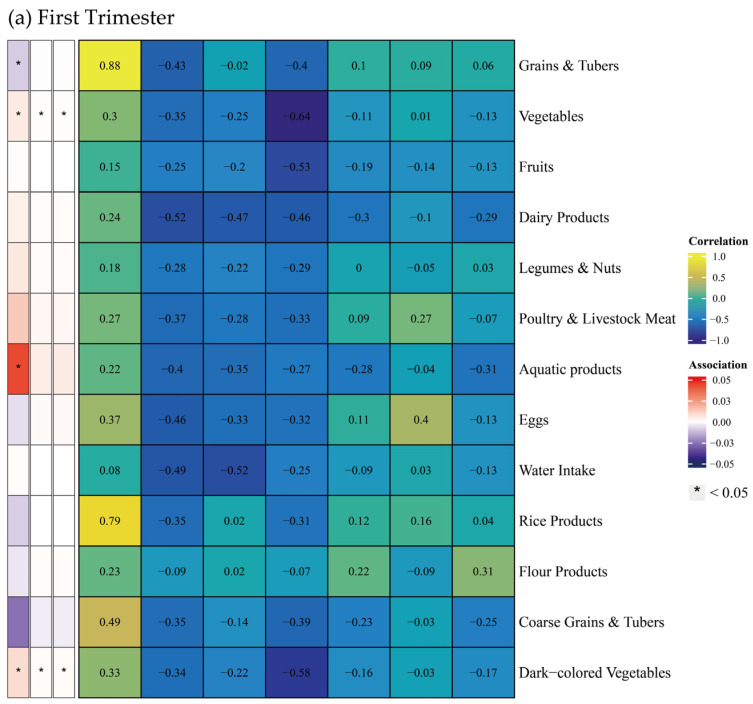

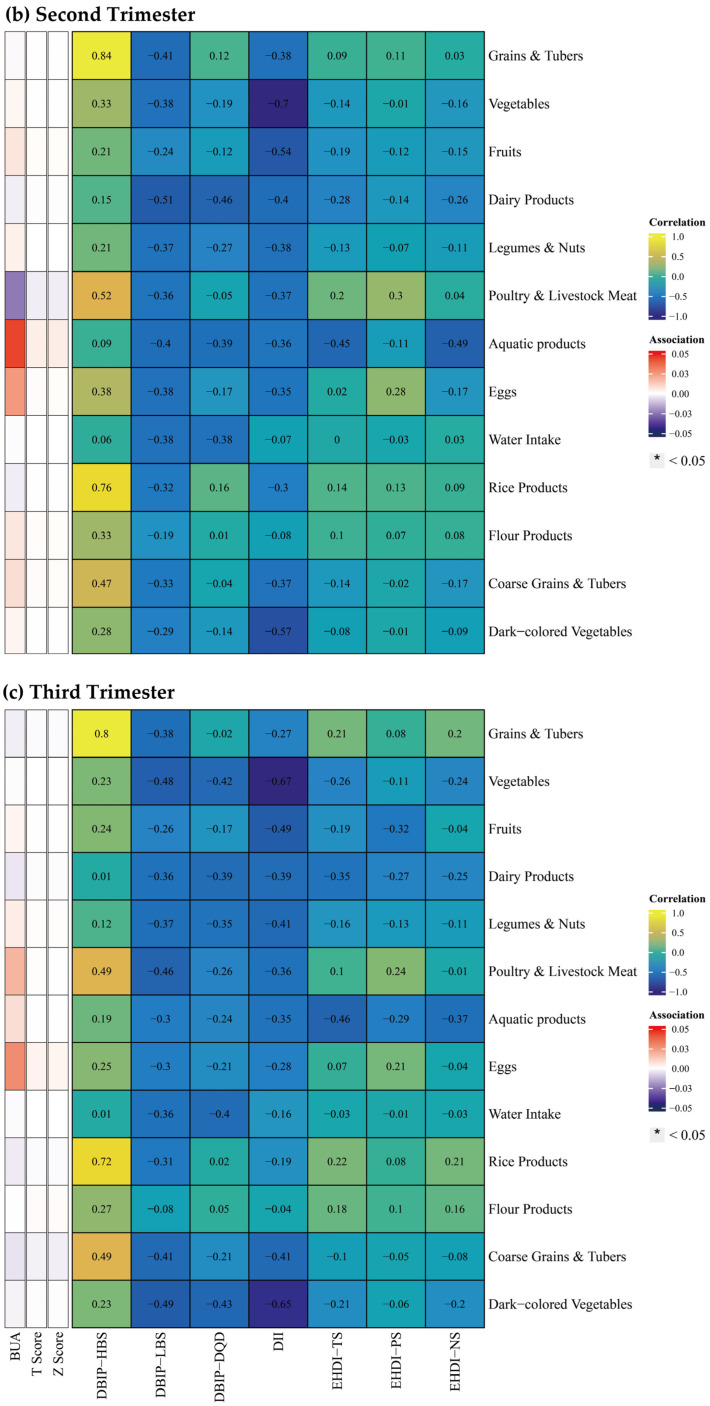
Spearman correlation between food groups and dietary indices, and multiple linear regression between food groups and bone mineral density across trimesters. Note: The left panel displays the results of the multiple linear regression model, with Spearman correlation results in the right panel. The multiple linear regression model was adjusted for age, gender, pre-pregnancy body mass index, education, income, gestational diabetes mellitus (GDM), light physical activity frequency and time, and sun exposure time. (**a**–**c**) Represents trimester-stratified results for correlations between food groups and dietary indices, and associations between food groups and bone mineral density.

Additionally, this study explored the correlations between food groups and BMD (Figure 6). During the first trimester, higher intake of vegetables, aquatic products, and dark-colored vegetables was positively correlated with BMD, while the intake of grains and tubers showed a negative correlation. No significant correlations between food groups and BMD were detected in second and third trimesters.

### 3.4. Mediating Effects of Body Composition on the Relationship Between Dietary Indices and BMD

Given the significant interplay observed between dietary, body composition parameters, and BMD during the first trimester, mediation analyses were conducted to explore the potential mediating effect of body composition on the relationship between dietary indices and BMD. Table 3 revealed that specific body composition parameters significantly mediated the correlations between dietary indices and BMD during the first trimester. Notably, each body composition parameter significantly mediated the relationship between DQD and BUA. Right upper limb muscle mass (ACME: β = −0.40, 95% CI: −0.92, −0.02) and extracellular water (ACME: β = −0.33, 95% CI: −0.82, −0.01) accounted for 19% and 18% of the variance in the DQD-BUA relationship, respectively.

### 3.5. Sensitivity Analyses

Compared with multiple linear regression models without adjustment for covariates, our results remained robust, confirming the stability of correlations between corresponding variables and outcomes across statistical approaches.

## 4. Discussion

Our findings demonstrate significant correlations between dietary indices and body composition during the first and second trimesters, including fluid compartments, protein, inorganic salt, muscle mass, and fat mass. These results indicated that an unbalanced, pro-inflammatory, and lower-quality diet might contribute to a decline in body composition across trimesters, except for the third trimester. With the trimester increased, the nutritional demand and basal metabolic rate grow [21,22], indicating increased maternal metabolic demands and energy allocation toward fetal growth during later gestation, thus weakening the correlations between dietary indices and body composition.

Notably, the differential effects of food groups on body composition highlight that dietary indices capture holistic dietary impacts rather than mere additive effects of individual foods. Poultry and livestock meat intake emerged as a critical dietary factor influencing body composition across trimesters, underscoring the importance of prioritizing these protein sources in prenatal nutrition. Prior evidence demonstrated that alterations in body composition parameters (e.g., excessive fat, altered fluid compartments) are linked to adverse outcomes, including GDM [23], gestational hypertension [24], and fetal growth abnormalities [25,26,27]. Our findings elucidate how maternal dietary patterns modulate gestational body composition trajectories. Given the established associations between altered maternal body composition and pregnancy complications, dietary optimization represents a plausible preventive strategy worthy of clinical investigation [25,26,27].

Dietary indices also exhibited trimester-specific correlations with BMD. During the first trimester, unbalanced dietary intake (excessive or insufficient intake) was correlated with reduced BMD. This aligns with evidence that early pregnancy demands heightened intestinal calcium absorption to meet fetal skeletal needs [28,29], especially in poor diet quality. Additionally, no significant correlations between dietary indices and BMD were detected in later trimesters, sustained attention to maternal nutrition remains critical, as fetal bone development peaks during these periods.

Furthermore, different dietary indices (DBI-P, DII, and EHDI) represent distinct dietary patterns, with varying effects on body composition and BMD. Aligned with the Chinese Dietary Guidelines, DBI-P showed robust correlations with body composition and BMD, due to its regional relevance and comprehensive reflection of balanced nutrient intake in China. This dietary pattern might be consistent with the diet of pregnant women in China, enabling them to reflect the characteristics of the diet. Representing the inflammation-related dietary pattern, DII strongly correlated with body composition changes, consistent with the role of systemic inflammation in modulating metabolic and compositional adaptations. Consequently, EHDI represents the Eastern coastal dietary pattern with diverse food and less culinary oil. It is the first time that the dietary evaluation utilizes an Eastern healthy dietary pattern, which also demonstrates the potential effect on body composition. EHDI demonstrated narrower applicability but unique value as a novel tool for assessing pregnancy-related dietary effects, warranting further validation.

This study has several limitations. Firstly, dietary data collected via FFQ may be susceptible to recall bias, potentially affecting intake accuracy. Moreover, while linear models are adjusted for various confounders, potential factors might not be included. Thirdly, cross-sectional research inherently limits causal inference regarding the observed relationships between dietary patterns, body composition, and bone mineral density. The absence of longitudinal data also limits conclusion about plausible mechanistic pathways from mediation model. Future prospective cohort studies should be conducted to validate our results. While the gestational milieu shapes the observed diet–body composition relationships, our analytical framework and pathological parallels may inform future studies in disparate clinical populations and objective biomarkers to strengthen causal inference.

## 5. Conclusions

This study demonstrates that early pregnancy represents a critical window during which maternal dietary patterns significantly modulate gestational body composition parameters and BMD. In the present study, the three dietary indices showed significant associations with body composition, especially in the first and second trimesters, suggesting that an unbalanced, pro-inflammatory, and low-quality diet contributed to a decline in body composition and indicated a negative correlation between DBI-P and BMD in the first trimester. In addition, body composition parameters explain 16–19% of dietary effects on BMD, revealing a previously unquantified physiological mechanism. Further research is warranted to confirm these findings among more diverse populations.

## Figures and Tables

**Table 1:** Comparison of three dietary assessment indices [8,9,10,11,12,13].

Feature	DBI-P	DII	EHDI
Core principle	Quantifies adherence to gestational nutritional recommendations by assessing dietary balance across food groups.	Quantifies the overall inflammatory potential of an individual’s diet based on the inflammatory properties of specific nutrients/foods.	Measures adherence to a traditional East Asian dietary pattern.
Development basis	Chinese Dietary Guidelines for Pregnant Women	Research on associations between dietary components and inflammatory biomarkers	Epidemiological evidence on traditional East Asian diets
Scoring method	Assigns scores for insufficiency/adequacy/excess of food groups relative to pregnancy targets.	Scores dietary components based on their inflammatory effects.	Scores intake levels of predefined food groups against recommended thresholds.
Key strengths	1. Pregnancy-specific targets and validation. 2. Identifies both deficiencies and excesses. 3. Directly informs clinical dietary counseling for pregnancy. 4. Culturally adapted for the Chinese population.	1. Mechanistic focus on inflammation. 2. Standardized methodology permits cross-population comparison. 3. Strong epidemiological associations with inflammation-related diseases.	1. Cultural specificity for East Asian populations. 2. Simpler application than nutrient-based indices.
Key limitations	1. Limited generalizability outside pregnancy or the Chinese population. 2. Requires detailed dietary data	1. Relies on existing inflammatory biomarker studies. 2. Requires comprehensive dietary data for all components.	1. May not fully address nutrient adequacy or balance. 2. Less validated for methodology.

**Table 2:** Distribution of demographic, dietary, body composition and BMD characteristics across trimesters in study participants.

Characteristic	First Trimester (*n* = 211)	Second Trimester (*n* = 184)	Third Trimester (*n* = 161)	*p* Value
Age (years)				0.379
≤25	40 (19.0)	29 (15.8)	16 (9.9)	
26~30	103 (48.8)	96 (52.2)	85 (52.8)	
31~35	44 (20.9)	38 (20.7)	41 (25.5)	
≥35	24 (11.4)	21 (11.4)	19 (11.8)	
Pre-pregnancy BMI (kg/m^2^)				0.752
<18.5	29 (13.7)	25 (13.6)	25 (15.5)	
18.5~24	147 (69.7)	122 (66.3)	112 (69.6)	
>24	35 (16.6)	37 (20.1)	24 (14.9)	
Education Level				0.32
Bachelor’s Degree and Above	90 (42.7)	78 (42.4)	73 (45.3)	
College/Technical College	56 (26.5)	62 (33.7)	51 (31.7)	
High School/Technical School and Below	65 (30.8)	44 (23.9)	37 (23.0)	
Ethnicity				0.287
Han	204 (96.7)	172 (93.5)	151 (93.8)	
Other	7 (3.3)	12 (6.5)	10 (6.2)	
Average Monthly Household Income (CNY)				0.42
≤6000	105 (49.8)	83 (45.1)	64 (39.8)	
6001~8000	35 (16.6)	37 (20.1)	32 (19.9)	
>8000	55 (26.1)	43 (23.4)	49 (30.4)	
Not Specified	16 (7.6)	21 (11.4)	16 (9.9)	
Occupation				0.373
Office Workers and Related Staff	44 (20.9)	31 (16.8)	24 (14.9)	
Unemployed	27 (12.8)	23 (12.5)	21 (13.0)	
Housekeeping	16 (7.6)	16 (8.7)	20 (12.4)	
Other Laborers	17 (8.1)	20 (10.9)	14 (8.7)	
Business and Service Industry Workers	53 (25.1)	43 (23.4)	27 (16.8)	
Professional and Technical Personnel	54 (25.6)	51 (27.7)	55 (34.2)	
Light Physical Activity Frequency				0.062
Less (<2 times/week)	37 (17.5)	21 (11.4)	12 (7.5)	
Moderate (<6 times/week)	58 (27.5)	57 (31.0)	53 (32.9)	
More (>1 time/day)	116 (55.0)	106 (57.6)	96 (59.6)	
Light Physical Activity Time				0.002
<30 min/day	100 (47.4)	71 (38.6)	52 (32.3)	
30~60 min/day	77 (36.5)	96 (52.2)	86 (53.4)	
>1 h/day	34 (16.1)	17 (9.2)	23 (14.3)	
Sun Exposure Time				0.108
<1 h/day	96 (45.5)	84 (45.7)	66 (41.0)	
1~2 h/day	77 (36.5)	81 (44.0)	75 (46.6)	
>2 h/day	38 (18.0)	19 (10.3)	20 (12.4)	
Gestational diabetes mellitus				<0.001
Yes	1 (0.5)	10 (5.4)	46 (28.6)	
No	210 (99.5)	174 (94.6)	115 (71.4)	
Body composition and bone density				
Intracellular Water	18.12 (1.98)	18.94 (1.97)	20.40 (2.34)	<0.001
Extracellular Water	11.27 (1.19)	11.99 (1.21)	12.92 (1.43)	<0.001
Total Body Water	29.40 (3.16)	30.92 (3.17)	33.32 (3.75)	<0.001
Protein	7.83 (0.86)	8.19 (0.85)	8.82 (1.02)	<0.001
Inorganic Salt	2.88 (0.30)	3.08 (0.30)	3.33 (0.36)	<0.001
Muscle Mass	37.71 (4.06)	39.62 (4.07)	42.69 (4.83)	<0.001
Right Upper Limb Muscle Mass	1.90 (0.35)	2.02 (0.34)	2.27 (0.39)	<0.001
Right Lower Limb Muscle Mass	6.15 (0.77)	6.39 (0.78)	6.85 (0.90)	<0.001
Left Upper Limb Muscle Mass	1.87 (0.35)	2.00 (0.34)	2.26 (0.40)	<0.001
Left Lower Limb Muscle Mass	6.15 (0.76)	6.38 (0.79)	6.83 (0.87)	<0.001
Trunk Muscle Mass	17.69 (2.07)	18.29 (2.09)	19.77 (2.35)	<0.001
Body Fat Mass	17.78 (6.03)	19.13 (5.70)	22.71 (6.06)	<0.001
Right Upper Limb Fat Mass	1.23 (0.52)	1.32 (0.50)	1.62 (0.59)	<0.001
Right Lower Limb Fat Mass	2.77 (0.83)	3.01 (0.82)	3.50 (0.89)	<0.001
Left Upper Limb Fat Mass	1.25 (0.52)	1.35 (0.49)	1.65 (0.60)	<0.001
Left Lower Limb Fat Mass	2.76 (0.82)	2.99 (0.81)	3.48 (0.88)	<0.001
Trunk Fat Mass	8.73 (3.29)	9.40 (3.05)	11.31 (3.11)	<0.001
BUA	34.81 (11.12)	33.75 (9.89)	32.78 (9.97)	0.173
T-Score	−0.37 (1.42)	−0.35 (1.34)	−0.58 (1.29)	0.221
Z-score	−0.15 (1.43)	−0.12 (1.33)	−0.34 (1.31)	0.267
Dietary characteristic				
Grains and Tubers	322.20 (189.65)	353.71 (213.40)	317.89 (228.28)	0.206
Vegetables	504.02 (379.41)	595.41 (444.75)	612.70 (475.58)	0.029
Fruits	313.38 (259.64)	335.58 (222.61)	318.47 (410.12)	0.752
Dairy Products	208.09 (186.08)	243.33 (197.05)	265.47 (236.76)	0.025
Legumes and Nuts	101.62 (129.02)	109.03 (130.16)	128.06 (151.62)	0.171
Poultry and Livestock Meat	54.85 (64.30)	70.92 (61.82)	67.72 (56.72)	0.023
Aquatic products	29.69 (43.13)	30.76 (30.89)	37.06 (50.06)	0.208
Eggs	43.39 (33.25)	52.01 (32.03)	59.48 (43.56)	<0.001
Water Intake	1257.75 (827.81)	1339.72 (764.00)	1402.32 (740.03)	0.203
Rice Products	225.56 (157.50)	249.50 (172.40)	225.14 (197.22)	0.31
Flour Products	49.46 (63.64)	48.01 (46.43)	42.26 (47.96)	0.42
Coarse Grains and Tubers	47.18 (57.30)	56.21 (69.48)	50.50 (54.00)	0.335
Dark-colored Vegetables	293.90 (232.60)	357.21 (299.40)	372.13 (312.47)	0.015

Note: Values are mean (SD) or *n* (%).

**Table 3:** Bootstrap analysis of the mediating effect of body composition between DQD and BUA.

	ACME	ADE	Total	Proportion
Intracellular Water	−0.41 (−0.99, −0.04)	−2.15 (−4.09, −0.35)	−2.57 (−4.67, −0.7)	0.16 (0, 0.74)
Extracellular Water	−0.33 (−0.82, −0.01)	−2.24 (−4, −0.58)	−2.57 (−4.42, −0.86)	0.18 (0.01, 0.7)
Total Body Water	−0.39 (−0.91, −0.02)	−2.18 (−3.9, −0.33)	−2.57 (−4.33, −0.69)	0.16 (0.02, 0.6)
Protein	−0.42 (−0.97, −0.03)	−2.15 (−3.91, −0.28)	−2.57 (−4.39, −0.74)	0.13 (0, 0.41)
Inorganic Salt	−0.48 (−1.04, −0.07)	−2.08 (−3.87, −0.35)	−2.57 (−4.5, −0.74)	0.15 (0.01, 0.56)
Muscle Mass	−0.4 (−0.92, 0)	−2.17 (−3.87, −0.27)	−2.57 (−4.4, −0.65)	0.16 (0.01, 0.6)
Right Upper Limb Muscle Mass	−0.4 (−0.92, −0.02)	−2.17 (−4.12, −0.31)	−2.57 (−4.46, −0.6)	0.19 (0.03, 0.59)
Left Upper Limb Muscle	−0.48 (−1.08, −0.07)	−2.08 (−3.93, −0.24)	−2.57 (−4.47, −0.71)	0.15 (0, 0.58)
Trunk Muscle Mass	−0.46 (−1.06, −0.07)	−2.1 (−4, −0.3)	−2.57 (−4.5, −0.77)	0.16 (0, 0.57)

Abbreviation: ACME, average causal mediation effects; ADE, average direct effects. Note: Adjusted for age, gender, pre-pregnancy body mass index, education, income, gestational diabetes mellitus (GDM), light physical activity frequency and time, and sun exposure time.

## Data Availability

The data presented in this study are available on request from the corresponding author. The data are not publicly available due to the further article publication.

## Abbreviations

The following abbreviations are used in this manuscript:

BMD	Bone mineral density
BUA	Broadband ultrasound attenuation
DBI-P	Dietary Balance Index for Pregnant women
DII	Dietary Inflammatory Index
DQD	Dietary Quality Distance
EHDI	Eastern Health Diet Index
FFQ	Food frequency questionnaire
GDM	Gestational diabetes mellitus
HBS	High Bound Score
LBS	Low Bound Score
NS	Negative Score
PS	Positive Score
TS	Total Score
USDA	United States Department of Agriculture
